# Is there a diagnostic benefit of late-phase abdomino-pelvic PET/CT after urination as part of whole-body ^68^ Ga-PSMA-11 PET/CT for restaging patients with biochemical recurrence of prostate cancer after radical prostatectomy?

**DOI:** 10.1186/s13550-022-00885-z

**Published:** 2022-03-04

**Authors:** Janna Morawitz, Julian Kirchner, Johannes Hertelendy, Christina Loberg, Lars Schimmöller, Mardjan Dabir, Lena Häberle, Eduards Mamlins, Christina Antke, Christian Arsov, Gerald Antoch, Lino M. Sawicki

**Affiliations:** 1 Department of Diagnostic and Interventional Radiology, Medical Faculty, University Dusseldorf, Moorenstrasse 5, 40225 Düsseldorf, Germany; 2 Department of Nuclear Medicine, Medical Faculty, University Dusseldorf, 40225 Düsseldorf, Germany; 3 Institute of Pathology, Medical Faculty, Heinrich-Heine-University and University Hospital Duesseldorf, Duesseldorf, Germany; 4 Department of Urology, Medical Faculty, University Dusseldorf, 40225 Düsseldorf, Germany

**Keywords:** PSMA, PET/CT, Prostate cancer, Biochemical recurrence, Lesion detection

## Abstract

**Background:**

To assess the diagnostic value of an additional late-phase PET/CT scan after urination as part of ^68^ Ga-PSMA-11 PET/CT for the restaging of patients with biochemically recurrent prostate cancer (BCR).

**Methods:**

This retrospective trial included patients with BCR following radical prostatectomy, who underwent standard whole-body early-phase PET/CT performed 105 ± 45 min and an additional late-phase PET/CT performed 159 ± 13 min after injection of ^68^ Ga-PSMA-11. Late-phase PET/CT covered a body volume from below the liver to the upper thighs and was conducted after patients had used the bathroom to empty their urinary bladder. Early- and late-phase images were evaluated regarding lesion count, type, localisation, and SUVmax. Reference standard was histopathology and/or follow-up imaging.

**Results:**

Whole-body early-phase PET/CT detected 93 prostate cancer lesions in 33 patients. Late-phase PET/CT detected two additional lesions in two patients, both local recurrences. In total, there were 57 nodal, 28 bone, and 3 lung metastases, and 7 local recurrences. Between early- and late-phase PET/CT, lymph node metastases showed a significant increase of SUVmax from 14.5 ± 11.6 to 21.5 ± 17.6 (*p* = 0.00007), translating to a factor of + 1.6. Benign lymph nodes in the respective regions showed a significantly lower increase of SUVmax of 1.4 ± 0.5 to 1.7 ± 0.5 (*p* = 0.0014, factor of + 1.2). Local recurrences and bone metastases had a SUVmax on late-phase PET/CT that was + 1.7 and + 1.1 times higher than the SUVmax on early-phase PET/CT, respectively.

**Conclusion:**

In patients with BCR following radical prostatectomy, an additional abdomino-pelvic late-phase ^68^ Ga-PSMA-11 PET/CT scan performed after emptying the urinary bladder may help to detect local recurrences missed on standard whole-body ^68^ Ga-PSMA-11 PET/CT. Lymph node metastases show a higher SUVmax and a stronger increase of SUVmax than benign lymph nodes on late-phase PET/CT, hence, biphasic ^68^ Ga-PSMA-11 PET/CT might help to distinguish between malignant and benign nodes. Bone metastases, and especially local recurrences, also demonstrate a metabolic increase over time.

## Introduction

BCR of prostate cancer is defined as two consecutive prostate-specific antigen (PSA) levels > 0.2 ng/ml after radical prostatectomy [1]. Depending on the guideline, imaging for biochemical recurrence includes abdomino-pelvic computed tomography (CT) and bone scintigraphy [2, 3]. But these conventional imaging methods are poor at detecting site of disease, especially at low PSA-levels [4–7]. The sensitive and accurate detection and localisation of site of disease is required to evaluate if BCR is reflecting local or distant progression and to drive therapy management decisions for treatment options of resection, targeted radiation or systemic therapy [3]. Nowadays a well-established imaging method is prostate specific membrane antigen (PSMA) positron emission tomography/computed tomography (PET/CT). PSMA is a transmembrane surface enzyme that shows a 100 to 1000-fold increase in expression on prostate cancer cells [8]. ^68^ Ga-PSMA-11 represents a ligand that binds highly specific to prostate carcinoma cells. While previous tracers such as ^18^F- or ^11^C-choline, ^1^C-acetate or ^18^F-fluciclovine were used but only showed moderate sensitivity [9–11], ^68^ Ga-PSMA has been shown to be well suitable for the detection of site of disease in BCR patients [12]. The current EAU Guidelines recommend PSMA PET/CT in BCR after radiotherapy in patients suitable for curative salvage therapy, or after radical prostatectomy, in patients with PSA > 0.2 ng/ml if the results are expected to influence the therapy decision [13]. However ^68^ Ga-PSMA-11 is eliminated via the urinary system and therefore accumulates in the urinary bladder. At the standard imaging time 100 min after injection, this can lead to obscuration of local recurrence in the prostatic fossa by overlaying activity in the urinary bladder. Hence, an additional late PET/CT scan after emptying the urinary bladder may eliminate this problem, as the radioactivity in the urinary bladder is massively reduced. In addition, it has been shown for multiple cancer entities that biphasic PET/CT entails various diagnostic benefits, such as the ability to assess standardized uptake value (SUV) dynamics, and is particularly suitable for distinguishing benign from malignant lesions [14, 15]. Therefore the aim of our study was to evaluate the diagnostic value of an additional late-phase abdomino-pelvic PET/CT scan after urinary bladder emptying as part of ^68^ Ga-PSMA-11 PET/CT for the restaging of patients with newly documented BCR of prostate cancer after radical prostatectomy.

## Material and methods

### Patients

The local Ethics Committee approved this retrospective study (Study-No.: 871-2020). Inclusion criteria were biochemical recurrence of prostate cancer (PSA-value of ≥ 0.2 ng/ml after radical prostatectomy with subsequent confirmatory test of PSA-value showing persistence of PSA-value ≥ 0.2 ng/ml). Lack of adequate reference standard, age < 18 years, or contraindications against iodinated contrast agents or ^68^ Ga-PSMA-11 were exclusion criteria. All PET/CTs performed were clinically indicated. Twenty-five patients of the study cohort had been reported in articles before [16, 17].

### PET/CT

Standard whole-body early-phase PET/CT was performed on a Biograph mCT 128 (Siemens Healthineers, Erlangen, Germany) 105 ± 45 min post injection of a weight adapted dose of ^68^ Ga-PSMA-11 (2 mg/kg bodyweight). Image acqusition was performed in supine position from skull base to mid thigh or from head to feet. The whole-body CT component was performed 70 s after intravenous injection of a weight-adapted dose of iodinated contrast agent (Accupaque 300, GE Healthcare, Munich, Germany). Additionally a low-dose deep inspiration chest CT scan was performed for better assessment of the lung tissue.

The late-phase PET/CT scan covered a body volume from just below the liver to the mid thighs and was conducted 159 ± 13 min after radiotracer injection, and after patients had emptied their urinary bladder. For urinary bladder emptying, patients were required to use the bathroom for emiction immediately after the whole-body early-phase PET/CT scan was finished. Patients were, without any avoidable delay, then repositioned on the PET/CT scanner in order to start the late-phase PET/CT scan. No additional iodinated CT contrast agent was injected for the late-phase PET/CT.

The following imaging parameters were used in both the early- and late-phase PET/CT: 120 kV, automatic mA/s adjustment (CARE Dose4D™, pre-set 210 mAs), slice thickness 2 mm, collimation 128 × 0.6 mm, pitch 0.8). PET data were acquired for 3 min in each bed position (matrix size 256 × 256, axial field of view 21.8 cm and a Gaussian filter of 4 mm). Standard CT-based attenuation correction was performed and iterative reconstruction using ordered subsets expectation maximization was applied with 3 iterations and 21 subsets.

### Image analysis

PET/CT datasets were evaluated on a Sectra-Workstation (IDS7; Sectra, Linköping, Sweden). The early- and late-phase PET/CT datasets were each separately and independently analysed by two radiologists (J. M.; J. K.) and two nuclear medicine specialists (M. D.; E. M.) with multiple years of experience in hybrid imaging. To avoid recognition bias, the data sets were read at least 4 weeks apart. Discrepancies were resolved in a consensus reading by an expert reader (L. M. S.) with 8 years of experience in PSMA PET/CT imaging. Readers were blinded to previous or follow-up imaging. The readers determined the location, quantity and type of lesion (local recurrence, lymph node metastasis, bone metastasis, etc.) for each patient individually. According to previous publications, lymph nodes with a short axis diameter > 8 mm (pelvic) or > 10 mm (inguinal, abdominal, thoracic, neck) were classified as morphologically suspicious. In addition, the malignancy criteria of spherical configuration, increased contrast enhancement and inhomogeneity were applied [18]. With regard to the PET data set, a visually focal tracer uptake above the surrounding background was considered as suspicious. To quantify tracer uptake by SUVmax, a three-dimensional region of interest was manually drawn around each suspicious lesion on early- and late-phase PET/CT. In all patients with suspicious lymph nodes, at least one non-suspicious, benign lymph node in the respective contralateral body region (i.e. contralateral para-iliac, contralateral inguinal etc.) was also evaluated by SUVmax. To measure the background SUVmax, a three-dimensional region of interest was placed in the M. gluteus maximus on early- and late-phase PET/CT.

### Reference standard

Whenever available, histopathological results were used as a reference standard. A surrogate reference standard was used for all those lesions that were not assessed histopathologically. The surrogate reference standard was based on adequate, clinically indicated follow-up imaging, such as follow-up PSMA PET/CT, scintigraphy, CT, and magnet resonance imaging (MRI). For any lesion reported in the image analysis, at least one of the above-mentioned reference standards was available. Moreover, a decreasing PSA value as well as a decrease in size and/or tracer uptake of lesions after therapy was considered as a sign of malignancy. In addition, lesions with increasing size and/or increasing tracer uptake in the course of therapy were considered malignant.

### Statistics

Statistical analysis was performed using SPSS version 26 (IBM, Armonk, NY, USA). A paired t-test was used to compare SUV-values between early- and late-phase PET/CT scans and to compare lesion/background ratios for benign and malignant lesions. A *p* value < 0.05 was considered as indicating statistical significance. Mean values are presented as average values ± standard deviation.

## Results

### Patients and reference standard

From a total of 33 eligible patients (mean age 73.5 ± 8.0 years), PSMA PET/CT was performed from head to feet in 19 patients and from base of the skull to mid thighs in 14 patients. Mean PSA at the time of imaging was 6.6 ± 11.0 ng/ml (range 0.27–50.0 ng/ml). According to the reference standard, in the 33 patients, a total of 95 lesions were detected, of which 57 were lymph node metastases, 28 were bone metastases, 7 were local recurrences and 3 were pulmonary metastases. Eight out of the 95 lesions were proven histopathologically. As described above, the reference standard for the remaining 87 lesions was prior and/or follow-up imaging: MRI in 4 patients, CT in 7 patients, PET/CT in 17 patients and decrease of PSA after radiation therapy in 2 patients.

### Early-phase PET/CT

Standard early-phase PET/CT was performed 105 ± 45 min after injection of a mean activity of 159 ± 37 MBq ^68^ Ga-PSMA-11. According to the reference standard, whole-body early-phase PET/CT detected 93/95 lesions (97.9%). In detail, early-phase PET/CT detected 57/57 (100%) lymph node metastases, 28/28 (100%) bone metastases, 5/7 (71.4%) local recurrences and 3/3 (100%) pulmonary metastases. All detected lesions were PET-positive. Average SUVmax of all detected lesions in early-phase PET/CT was 15.9 ± 12.5. In detail, SUVmax for lymph node metastases, bone metastases, local recurrences and pulmonary metastases was 14.5 ± 11.6, 16.2 ± 15.3, 8.8 ± 7.4 and 8.6 ± 11.2, respectively (Table 1). Two local recurrences were missed by early-phase PSMA PET/CT, but were detected by late-phase PSMA PET/CT.

**Table 1:** Lesions detected in early-phase PET/CT

	Lesion count	SUVmax	Lesion/background ratio
Total	93	15.9 ± 12.5	23.6 ± 21.8
Lymph node metastasis	57	14.5 ± 11.6	25.5 ± 19.4
Bone metastasis	28	16.2 ± 15.3	22.4 ± 27.6
Local recurrence	5	8.8 ± 7.4	17.0 ± 9.8
Pulmonary metastasis	3	8.6 ± 11.2	14.3 ± 20.3

On early-phase PET/CT the SUVmax/background ratio for the total of 54 benign lymph nodes was 2.2 ± 1.1, whereas the SUVmax/background ratio for the corresponding lymph node metastases was 25.5 ± 19.4. Differences between these ratios were statistically significant (*p* < 0.0001).

### Late-phase PET/CT

**Fig. 1:**
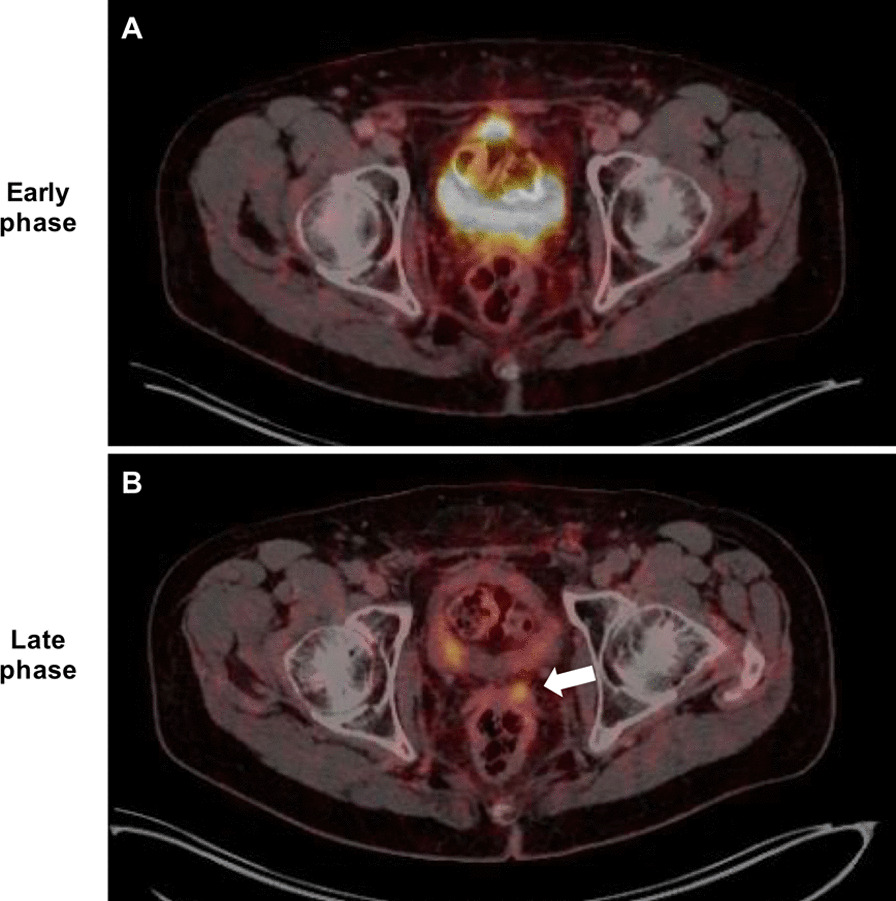
Example of a 72-year old patient with biochemical recurrence (PSA 1.76 ng/ml at the time of imaging). There was no tumorous lesion detectable on early-phase ^68^ Ga-PSMA PET/CT (**A**) because of obscuration due to the tracer in the urinary bladder. Late-phase ^68^ Ga-PSMA PET/CT performed after emptying of the urinary bladder (**B**) however, revealed the local recurrence in the seminal vesicles (white arrow)

**Fig. 2:**
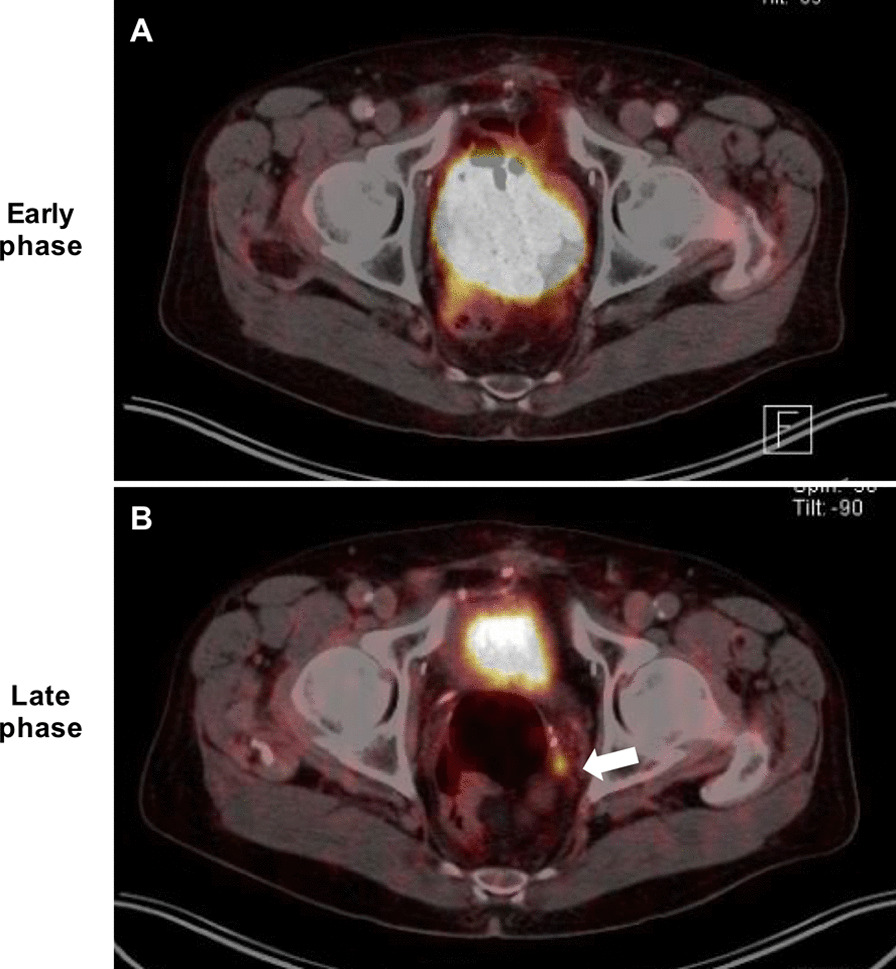
Example of a 81-year old patient with biochemical recurrence (PSA 1.5 ng/ml at the time of imaging. Para-rectal local recurrence (white arrow) was detected in late phase ^68^ Ga-PSMA PET/CT (**B**) because of enhanced tracer uptake (SUVmax 18.3), but was PET-negative in early phase^68^Ga-PSMA PET/CT (**A**). The suspected diagnosis of local recurrence was confirmed by follow-up multiparametric pelvic MRI

The delay between the standard whole-body early-phase PET/CT and the abdomino-pelvic late-phase PET/CT was 54 ± 32 min (i.e. early-phase: 105 ± 45 min after injection vs. late-phase: 159 ± 13 min after injection). According to the reference standard, abdomino-pelvic late-phase PET/CT detected 59/59 (100%) lesions. Fifty-seven of them were identical to the lesions detected by early-phase PET/CT, but late-phase PET/CT was able to identify two additional local recurrences in 2/33 (6.1%) patients, which were missed by standard early-phase PET/CT (Table 2). These two patients showed a negative PET/CT scan in early phase. One of the two missed local recurrences was located in the seminal vesicles. It was obscured by radiourine in the urinary bladder on early-phase PET images and too small to detect on morphological CT images (Fig. 1). The second missed local recurrence was also small, located in the para-rectal space and PET-negative on early-phase PET/CT. However, it showed a markedly enhanced tracer uptake on the late-phase PET/CT, in keeping with local recurrence (Fig. 2).

**Table 2:** Lesions detected in the scan-volume of late-phase (i.e. abdomino-pelvic only)

	Early phase		Late phase	
	Lesion count	SUVmax	Lesion count	SUVmax
Total lesions	57/59 (96.6%)	13.5 ± 10.9	59/59 (100%)	18.4 ± 16.0
Lymph node metastases	36/36 (100%)	14.8 ± 11.7	36/36 (100%)	21.5 ± 17.6
Bone metastases	16/16 (100%)	11.4 ± 10.2	16/16 (100%)	12.4 ± 11.1
Local recurrence	5/5 (71.4%)	8.8 ± 7.4	7/7 (100%)	17.7 ± 12.6

Of the 59 lesions detected on late-phase PET/CT, 36 were lymph node metastases, 16 were bone metastases and 7 were local recurrences. Average SUVmax of all detected lesions in late phase was 18.4 ± 16.0. For lymph node metastases, SUVmax was 21.5 ± 17.6, for bone metastases and local recurrences it was 12.4 ± 11.1 and 17.7 ± 12.6 respectively (Table 3). For the respective SUVmax of the lesions in the bodyvolume of the late phase scan (i.e. abdomino-pelvic only) in early phase see Table 2.

**Table 3:** Lesions detected in late-phase PET/CT

	Lesion count	SUVmax	Lesion/background ratio
Total	59	18.4 ± 16.0	22.9 ± 19.7
Lymph node metastasis	36	21.5 ± 17.6	27.7 ± 19.7
Bone metastasis	16	12.4 ± 11.1	15.1 ± 19.1
Local recurrence	7	17.7 ± 12.6	15.0 ± 10.8

On late-phase PET/CT the SUVmax/background ratio for the total of 34 benign lymph nodes was 2.3 ± 1.2, whereas the SUVmax/background ratio for the corresponding lymph node metastases was 27.7 ± 19.7. Again, differences between these ratios were statistically significant (*p* < 0.0001).

### SUV dynamic from early-phase to late-phase PET/CT

The SUVmax of lymph nodes metastases in the scan volume of late-phase (i.e. abdomino-pelvic only) was significantly higher on late-phase than on early-phase PET/CT: 21.5 ± 17.6 vs. 14.8 ± 11.7; *p* = 0.00003. Also, the SUVmax of benign lymph nodes was significantly higher on late-phase than on early-phase PET/CT: 1.7 ± 0.5 vs. 1.4 ± 0.5, *p* = 0.02.

**Fig. 3:**
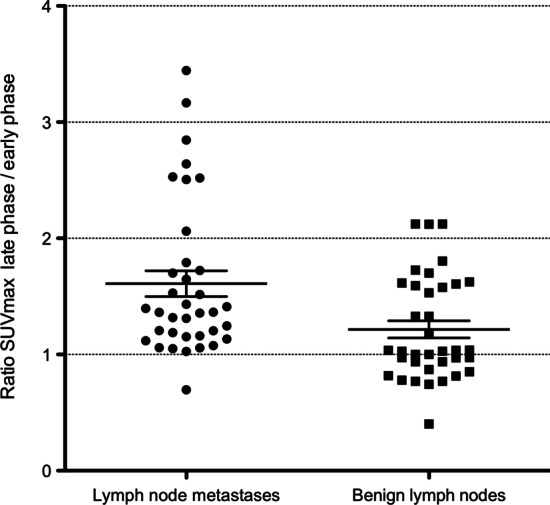
Late-phase/early-phase SUVmax ratio of lymph node metastases versus benign lymph nodes

The mean ratio between late-phase SUVmax and early-phase SUVmax of identical lymph node metastases was significantly higher than the respective ratio for benign lymph nodes (+ 1.6 ± 0.7 vs. + 1.2 ± 0.4, *p* = 0.0014) (Fig. 3).

The SUVmax of local recurrences and of bone metastases was significantly higher on late-phase than on early-phase PET/CT, respectively: in early-phase PET/CT local recurrences showed a SUVmax of 8.8 ± 7.4, in late-phase PET/CT 17.7 ± 12.6. For abdomino-pelvic bone metastases it was 11.4 ± 10.2 in early-phase PET/CT and 12.4 ± 11.1 in late-phase PET/CT. The mean ratio between late-phase SUVmax and early-phase SUVmax of local recurrences was + 1.7 ± 0.4. and + 1.1 ± 0.6 of bone metastases, respectively.

## Discussion

In this work, we showed that, in a small proportion of patients with BCR following radical prostatectomy, the acquisition of an additional late-phase abdomino-pelvic PET/CT as part of a biphasic whole-body ^68^ Ga-PSMA-11 protocol enables the detection of additional local recurrences, which are invisible on standard early-phase PET/CT. Moreover, on late-phase PET/CT the SUVmax of local recurrences is about 4-times higher than on standard early-phase ^68^ Ga-PSMA-11 PET/CT, which might be a helpful hallmark in order to differentiate them from post-prostatectomy scar tissue. We found that the assessment of SUVmax dynamics might even help to distinguish between benign and malignant lymph nodes, since the SUVmax of lymph node metastases is not only much higher but the increase of SUVmax over time is more pronounced in lymph node metastases than in benign lymph nodes. Conversely, a very low and decreasing SUVmax over time makes a lymph node metastasis highly unlikely. In contrast to lymph node metastases and local recurrences, bone metastases tend to show almost no increase of SUVmax between the early-phase and late-phase PET/CT.

Since therapeutic approaches to BCR vary widely, ranging from systemic therapy to salvage surgery to targeted irradiation, it is critical to accurately determine the site and burden of disease. Some guidelines like the European Association of Urology or the American Urological Association already endorse the use of PSMA PET/CT for the staging of patients with newly documented BCR [3, 19]. One advantage of ^68^ Ga-PSMA PET/CT over other tracers such as ^11^C-Choline or ^18^F-Choline is that it does not require any specific patient preparation in the form of a 6-h fast before the examination. In clinical routine, the PET/CT examination is usually performed 100 min after injection of ^68^ Ga-PSMA. Due to renal excretion, this leads to a pronounced accumulation of the tracer in the urinary bladder. Since local recurrences after radical prostatectomy occur mainly at the vesicourethral anastomosis, rectovesical space or the urinary bladder neck, the disadvantage of ^68^ Ga-PSMA due to accumulation of the tracer in the urinary bladder is well known in the literature [20, 21]. However, there are different ways to circumvent this problem: on the one hand, a transurethral urinary catheter can minimise the accumulation in the urinary bladder, on the other hand, the administration of diuretics can help. In addition, ^18^F-PSMA, a tracer that has reduced urinary clearance due to its biliary excretion, has recently been introduced [22–24].

In line with our results, Afshar-Oromieh et al. also found that the detection rate of PCa lesions is higher at a later time point compared to a scan performed at 1 h post-injection, especially when furosemide was used to reduce radioactivity in the bladder [25]. Another study by Alberts et al. also demonstrated a higher detection rate at a later time point, especially when diuretics were given before the late scan [26]. This is based on the improved tumor-to-background visibility at later time point images, and on the improved lesion-to-bladder contrast after diuretic administration. Here, however, it seems logical that even with a strong diuretic drug the lesion-to-bladder ratio cannot be improved as much as with a completely emptied bladder after urination. The role of multi-phasic ^68^ Ga-PSMA PET/CT for the differentiation of lymph node metastases from neural ganglia has also already been investigated [27]. Here, too, the value of an additional late-phase PET/CT scan could be demonstrated. The benefit of a late PET/CT scan was also demonstrated for other tracers such as ^18^F-DCFPyL [28]. Overall, several studies show that an increased tracer uptake on late-phase PET scan is typical for lymph node metastases, but the exact timing of the late scan differs widely in these studies. Also in this study, the time at which late-phase PET/CT was performed should not be considered a fixed time point, but rather the first time point after voiding of the bladder by urination. Hence, further studies are needed to determine the best time point to differentiate lymph node metastases from benign lesions. On the other hand, some studies investigated the use of an additional very early PET/CT scan performed 3 min after tracer injection, before the radiotracer is accumulating in the urinary bladder, and could demonstrate an improved accuracy [29, 30]. Overall, the current evidence suggests that the accumulation of ^68^ Ga-PSMA-11 in the bladder slightly limits the diagnostic performance of PET/CT and may, in some cases, impede the detection of local recurrences. In our study, the reduced radioactivity in the bladder—achieved by emptying the bladder before the late-phase scan—showed that additional local recurrences can be detected compared to standard early-phase PET/CT. In this study, bone metastases showed the smallest SUVmax increase from early- to late-phase. This is consistent with previous studies, showing that bone metastases of PCa can even show a decreasing tracer uptake over time [31]. The additional radiation exposure from the CT component of late-phase PET/CT, that was necessary because of the altered patient position after the patients had used the toilet, should not be neglected. However, the accumulated radiation exposure from diagnostic imaging in this age- and patient-cohort is generally of subordinate importance in regard to mortality or morbidity.

This study had limitations. First, the retrospective study design is prone to underestimate confounding factors. Second, the majority of the suspicious lesions could not be confirmed histopathologically due to ethical and clinical standards. Instead, a well-established reference standard consisting of adequate follow-up imaging and clinical parameters was availa ble for all lesions. Third, the patient population was relatively small. Especially, the low number of local recurrences may have limited the generalisability of the results. Therefore, generalized use of a late phase PET/CT scan might not be justified in all patients. Furthermore, only patients after radical prostatectomy were included, so that this study does not allow any statement about patients with biochemical recurrence after primary curative radiotherapy.

## Conclusion

In summary, additional late-phase abdomino-pelvic PET/CT, performed after patients have emptied their urinary bladder and as part of a biphasic whole-body ^68^ Ga-PSMA-11 protocol, offers several diagnostic advantages compared to mono-phasic, standard-phase ^68^ Ga-PSMA PET/CT, including the detection of otherwise occult local recurrences or to draw conclusions on the nature of lymph nodes and potential local recurrences based on the level and increase of SUVmax over time.

It is worth mentioning that in this study the additional local recurrences in the late phase were detected in patients with comparatively low PSA levels (1.76 ng/ml and 1.5 ng/ml vs. 6.6 ng/ml median in the study cohort), so it seems that especially in patients with low PSA-levels the additional late phase may be of importance. Additionally, the two patients in whom local recurrence was detected only in the late-phase scan were the only patients who had a completely negative early-phase scan. Therefore, it should be discussed, that an additional late phase should be performed only in patients with negative early phase. Nevertheless, further prospective studies with larger populations are needed to substantiate our findings.

## Data Availability

The datasets used and/or analysed during the current study are available from the corresponding author on reasonable request.

## Abbreviations

^11^C: Carbon-11
^18^F: Fluoride-18
^68^ Ga: Gallium-68
BCR: Biochemical recurrence
cm: Centimeter
CT: Computed tomography
kg: Kilogram
kV: Kilovolt
mAs: Miliampere seconds
MBq: Megabecquerel
mg: Miligram
min: Minutes
ml: Mililiter
mm: Milimeter
MRI: Magent resonance imaging
ng: Nanogram
No.: Number
PCa: Prostate cancer
PET/CT: Positron emission tomography/computed tomography (PET/CT)
PSA: Prostate specific antigen
PSMA: Prostate specific membrane antigen
SUV: Standardized uptake value
SUVmax: Maximum standardized uptake value
